# The processing of raising and nominal control: an eye-tracking study

**DOI:** 10.3389/fpsyg.2015.00331

**Published:** 2015-03-24

**Authors:** Patrick Sturt, Nayoung Kwon

**Affiliations:** ^1^Psychology, School of Philosophy, Psychology and Language Sciences, University of EdinburghEdinburgh, UK; ^2^Department of English Language, Konkuk UniversitySeoul, South Korea

**Keywords:** parsing, memory retrieval, eye-tracking, dependency formation, binding, raising, control

## Abstract

According to some views of sentence processing, the memory retrieval processes involved in dependency formation may differ as a function of the type of dependency involved. For example, using closely matched materials in a single experiment, Dillon et al. (2013) found evidence for retrieval interference in subject-verb agreement, but not in reflexive-antecedent agreement. We report four eye-tracking experiments that examine examine reflexive-antecedent dependencies, combined with raising (e.g., “John seemed to Tom to be kind to himself…”), or nominal control (e.g., “John's agreement with Tom to be kind to himself…”). We hypothesized that dependencies involving raising would (a) be processed more quickly, and (b) be less subject to retrieval interference, relative to those involving nominal control. This is due to the fact that the interpretation of raising is structurally constrained, while the interpretation of nominal control depends crucially on lexical properties of the control nominal. The results showed evidence of interference when the reflexive-antecedent dependency was mediated by raising or nominal control, but very little evidence that could be interpreted in terms of interference for direct reflexive-antecedent dependencies that did not involve raising or control. However, there was no evidence either for greater interference, or for quicker dependency formation, for raising than for nominal control.

## 1. Introduction

Successful language comprehension requires the computation of grammatical dependencies between linguistic elements in each sentence. For example, the interpretation of (1) requires a dependency between the reflexive *himself* and its antecedent *John*:

1. Bill thought that John was kind to himself.

However, although a great deal of research has been directed at the factors that affect processing difficulty during sentence comprehension, it is only recently that researchers have begun to turn their attention to the actual mechanisms that are used in on-line dependency formation.

One important aspect of dependency computation that has recently been examined in a number of studies is *memory retrieval*. Given that linguistic input is sequential, the two end-points of a dependency (e.g., *John* and *himself* in 1) are necessarily separated in time. In cases like (1), this means that memory access is required to solve the dependency—in order to interpret *himself*, the antecedent *John* needs to be retrieved from working memory. Recent work in human sentence processing has sought to examine the types of memory retrieval processes that best characterise linguistic dependency formation. According to a well-known view (e.g., McElree et al., 2003; Lewis and Vasishth, 2005; Lewis et al., 2006; Van Dyke and McElree, 2006), memory retrieval in sentence processing is a content-addressable process, in which potential targets in memory are activated in response to retrieval cues. For example, in (1), when *himself* is processed, the retrieval cues might include gender (the antecedent has to be masculine), as well as relevant structural information (the antecedent has to be in an appropriate local position relative to *himself*). According to such models, the activation of dependency targets is a parallel process, where multiple potential targets may be activated simultaneously through partial cue matching. This means that the retrieval of a grammatically licit retrieval target may be affected by the presence of other (grammatically illicit) items that partially match the retrieval cues, a phenomenon known as *interference*. For example, in (1), during the retrieval of the grammatically correct antecedent *John*, the grammatically illicit antecedent *Bill* may become partially activated, as it matches the *male* feature required by *himself*. This may affect the time taken to retrieve the correct antecedent *John*.

Computational models that make predictions concerning retrieval speed (e.g., Lewis and Vasishth, 2005; Lewis et al., 2006) predict that that interference can be either *facilitatory* (speeding up retrieval) or *inhibitory* (slowing down retrieval), depending on the contents of working memory at the point where retrieval takes place, and on the retrieval cues. These models assume a monotonic relation between retrieval times predicted by the model and reading times at the relevant point of the sentence where retrieval is assumed to occur. Below, we briefly describe two patterns of interference that have been reported in the literature. In this paper, we will refer to these as *facilitatory interference* and *inhibitory interference* respectively.

Facilitatory interference can be illustrated using the subject-verb agreement examples given in (2a,b), taken from the self-paced reading study reported by Wagers et al. (2009):

2a. The musician who the reviewer praise so highly will probably win a grammy.
2b. The musicians who the reviewer praise so highly will probably win a grammy.

Both (2a) and (2b) are ungrammatical, due to the number mismatch between the plural verb *praise* and the singular relative clause subject *reviewer*. However, Wagers et al. (2009) found that the reading time penalty was significantly reduced in (2b), which includes a plural distractor *the musicians*, relative to (2a), which does not. In this paper, we will use the term *facilitatory interference* specifically to refer to the reduction of processing difficulty (and thus faster retrieval) for a mismatching dependency, due to the presence of a partially matching distractor (see also Vasishth et al. (2008) and Xiang et al. (2009) for examples of facilitatory interference in other types of dependencies).

In the computational model proposed by Lewis and Vasishth (2005) and Lewis et al. (2006), facilitatory interference is explained in terms of mis-retrieval of the illicit retrieval target. For example, in (2a), the retrieval cues of the verb lead to activation of all potential targets in parallel, including both a licit and an illicit antecedent. However, the mismatching number feature on the licit antecedent, *the reviewer* in (2a,b) means that its retrieval takes a relatively long time. Similarly, in (2a), there is relatively little feature overlap between the distractor, *the musician* and the retrieval cues, leading to a lower activation of the distractor, and thus lower probability of misretrieval. On the other hand, in (2b) the distractor, *the musicians* partially matches the retrieval cues of the verb, sometimes leading to mis-retrieval of *the musicians* as the subject of *praise*. This “illusionary licensing” effect could lead to faster processing in (2b) relative to in (2a).

The second phenomenon that has been argued to follow from a content-addressable memory system is *inhibitory interference*. To illustrate this phenomenon, consider (3a,b), from Badecker and Straub (2002):

3a. John thought that Bill owed him another chance to solve the problem.
3b. John thought that Beth owed him another chance to solve the problem.

In both (3a) and (3b), the only grammatically licit antecedent for the pronoun *him* is *John*. However, in (3a), there is also a gender-matching (grammatically illicit) distractor (*Bill*), while (3b) contains a mismatching distractor *Beth*. Badecker and Straub (2002) found that the two words following the pronoun were read more slowly in (3a) than in (3b). In this paper, we use the term *inhibitory (retrieval) interference* to refer to processing difficulty (and thus slow retrieval) that occurs when the intended dependency target completely matches the retrieval cues (e.g., *John* in 3a), but where there is also a partial match with the distractor—for example, *Bill* in 3a is a distractor, as it is not a grammatically possible antecedent for *him*, but it partially matches the retrieval cue, as it bears the required *male* feature^1^.

In the computational model of Lewis and Vasishth (2005) and Lewis et al. (2006), inhibitory interference effects can be explained in terms of the parallel activation of all partially matching retrieval targets; in (3a), the distractor *Bill* has a relatively high activation level during the retrieval of *John*, due to the fact that it partially matches the features of the retrieval cue (i.e., it is masculine), and this leads to competition, slowing down the retrieval of the intended referent *John*. In contrast, in (3b), the distractor *Beth* overlaps to a lesser degree with the retrieval cue, leading to a relatively low activation, and thus less competition.

To summarize, in this paper, we use the term *facilitatory interference* to refer specifically to facilitation in the retrieval of a *feature mismatching* retrieval target; and we use *inhibitory interference* to refer speficically to inhibition in the retrieval of a *feature matching* retrieval target. In both cases, this is due to a the presence of a distractor that partially matches the retrieval cue. The previous literature on interference effects in dependency formation has yielded a mixed picture—although both facilitatory and inhibitory interference effects have been found, neither of these have been found consistently across different dependency types. For example, while Badecker and Straub (2002) found inhibitory interference for both pronoun-antecedent dependencies and reflexive-antecedent dependencies, these results have seldom been replicated. In fact, subsequent studies have failed to replicate inhibitory interference for both pronouns (Chow et al., 2014) and reflexives (e.g., Dillon et al., 2013, *inter alia*).

Facilitatory interference is reliably found for subject-verb agreement (e.g., Wagers et al., 2009) and negative polarity licensing (Vasishth et al., 2008; Xiang et al., 2009) but has not been consistently found for reflexive-antecedent agreement. Indeed, one recent study (Dillon et al., 2013) has directly compared these two dependency types in a single experiment, and found facilitatory interference effects only for subject-verb agreement, but no evidence for interference for reflexive-antecedent agreement.

The correct explanation for this variability in interference effects is not currently known. Concentrating on the variability of facilitatory interference across dependency types, Phillips et al. (2011) suggest that the parser may make use of either a structure-sensitive search process, or a content-addressable retrieval process, depending on the certain features of the dependency that is being computed: Specifically, Phillips et al. (2011) suggest that the type of memory access that is used may depend on how quickly structural information becomes available relative to other information.

Another possibility, argued by Dillon et al. (2013), is that all dependency types involve a content addressable retrieval process, but that the cues used for retrieval differ between different dependency types. This idea predicts that different types of dependency may lead to different interference profiles, even though they may target the same item in memory, for example, the subject of the local clause. For example, Dillon et al. (2013) contrasted reflexive-antecedent dependencies with subject-verb dependencies, both of which involve the local subject as a retrieval target. Based on their finding of facilitatory interference only in subject-verb dependencies, they argued that, while both dependencies make use of structural cues targeting the local subject, only subject-verb agreement uses the featural cue of number. The use of number as a retrieval cue in the subject-verb dependencies predicts that number-matching distractors become activated during retrieval, leading to interference, as observed by both Dillon et al. (2013) and others such as Wagers et al. (2009). In contrast, Dillon et al. (2013) argue that reflexive-antecedent dependencies only make use of structural retrieval cues, but not featural cues such as number and gender. If number is not used as a retrieval cue for reflexive-antecedent dependencies, this predicts that number-matching distractors are not activated during retrieval, which in turn predicts a lack of interference effects, in contrast with subject-verb agreement^2^.

The idea that different types of dependencies could involve different retrieval cues or processes, however, has not yet been tested using a wide range of dependencies. In particular, few studies have examined the retrieval processes of lexically-based dependencies, or compared them systematically with more structurally-based dependencies. Accordingly, it is not clear how retrieval processes would differ between these two types of dependencies. Thus, in this paper, we compared the retrieval processes of raising and nominal control constructions, which are illustrated in (4a,b) below.

4a. **Raising:**
  It was surprising that John seemed Ø to be kind to himself.
4b. **Nominal control:**
  I was surprised at John's agreement Ø to be kind to himself.

In (4a,b), the phonologically unexpressed subject of the infinitive (marked Ø in the above examples) participates in a dependency with its antecedent *John*. In (4a), the dependency is formed through raising, while in (4b), it is formed through nominal control. Raising and nominal control differ in many ways that could be relevant for processing. One important difference lies in the way that a dependency is motivated. That is, while the interpretation of raising is structurally constrained, the interpretation of nominal control depends crucially on lexical properties of the control nominal. For example, compare (5a) and (5b) below:

5a. John's agreement with Mary Ø to be kind to himself.
5b. John's order to Mary Ø to be kind to herself.

In (5a), the control nominal *agreement* is an instance of *giver control* (see Culicover and Jackendoff, 2001, for an overview of nominal control), meaning that Ø is interpreted as co-referential with the *giver* of the agreement (i.e., *John*)^3^. This leads to an interpretation in which *John* is kind to himself. In (5b), in contrast, *order* is an instance of *recipient control*, meaning that Ø is interpreted as co-referential with the *recipient* of the order (i.e., *Mary*). The interpretation is that *Mary* is kind to herself. In contrast with nominal control dependencies, raising dependencies, such as (4a) above do not exhibit lexically specific variability in the range of potential interpretations: if a raising verb (e.g., *seemed*) has a referential subject (e.g., *John* in 4a), then this must always be co-referential with the subject of the infinitive complement (i.e., Ø in 4a; cf. Hornstein, 1999). Thus, in (6) below, even though an “experiencer” distractor argument (i.e., *Mary*) intervenes, the raising construction still requires co-reference with *John*:

6. John seems to Mary Ø to be kind to himself.

These differences arguably have an analog in a representational distinction that syntacticians typically draw between raising and control. For example, in the Principles and Parameters framework (e.g., Chomsky, 1986) the empty subject in the raising example (6) is assumed to be an instance *NP-trace*, which participates in a strictly local and structurally constrained dependency with its antecedent. In contrast, the empty subject in all varieties of control, including nominal control (5), is assumed to be PRO, a pronominal element that is much less constrained, and whose choice of antecedent will depend on many factors, including the type of control relation. In other syntactic frameworks, the representational difference between (5) and (6) is even more marked—for example, in certain varieties of Lexicalized Tree Adjoining grammar (LTAG), the raising example (6) would not be assumed to include an empty infinitival subject at all^4^, while the control example (5) would include PRO, as in the Principles and Parameters framework (see the X-tag grammar of English, XTAG Research Group, 1998, for a framework that takes this approach). For the purposes of the present paper, we will continue to assume that both raising and nominal control involve an empty subject, but we will return to consider the predictions of the LTAG proposal where relevant below.

What types of cues might be used in retrieving the antecedent of the empty subject Ø in (5) and (6)? We assume that the empty subject is initially recognized around the point where *to be kind* is reached in the input, and we also assume that a retrieval process is launched around this point, to find the antecedent of Ø. Given the discussion above, it would make sense to assume that the retrieval cue for the raising dependency (6) would be structural in nature, (for example, targeting the subject of the next-highest finite clause). For nominal control (6), the retrieval cue would need to be represented in a more complex way, as it would need to refer to the control predicate (e.g., *agreement* or *order*), and locate the required target based on that predicate's control properties and argument structure.

In the studies reported in this paper, we examine the processing of sentences that are similar to (5) and (6), in that they combine a control or raising dependency with a reflexive-antecedent dependency. In both (5a) and (6), the dependency between the reflexive *himself* and its ultimate antecedent *John* is indirect—there is one (anaphor-antecedent) dependency between the reflexive and the empty subject, and another (raising or control) dependency between the empty subject and its antecedent. In other words, the dependency between the reflexive and its antecedent is mediated by nominal control (5) or raising (6). We therefore assume that the process of retrieval of the reflexive's antecedent is also mediated by nominal control or raising in cases like these (5) and (6). As a consequence, there are (at least) two retrieval events that involve raising or control in each of these sentences—the initial retrieval of the empty subject's antecedent around the infinitival verb, and a second retrieval, triggered by the reflexive. This second retrieval event, which is the focus of the experiments reported in this paper, has a wider range of cues that could potentially be relevant, because the reflexive provides gender and number information that is not available at the point where the empty infinitival subject is initially recognized—in the case of (5a) and (6), the reflexive requires its antecedent Ø to be male and singular, so Ø in turn must also require its antecedent to be male and singular. Whether each of these dependencies actually uses gender or number as retrieval cues is an empirical question. However, given that the nominal control dependency involves the element PRO, which is a species of pronoun, whose resolution is influenced by a wide range of factors, we believe that this dependency is more likely to use gender and number as a retrieval cue than the purely structural raising dependency.

In this paper, we test the hypothesis that nominal control dependencies would be (a) more prone to interference, and (b) processed more slowly, than raising dependencies. There are several reasons why nominal control dependencies might be expected to be more susceptible to interference than raising dependencies. One reason is that, as discussed above, the resolution of nominal control dependencies requires the use of complex constraints involving lexical information, while raising dependencies can be resolved through purely structural means. This might lead to more indeterminacy in the retrieval process for nominal control, leading to more interference, or it might mean that the two dependency types use qualitatively different retrieval mechanisms, for example, an interference-prone content-addressable mechanism for nominal control but a direct structure-based search for raising. A second possible reason is that, even if both dependency types use a content-addressable mechanism, nominal control dependencies may use a wider array of retrieval cues than raising dependencies, allowing more opportunity for a partial match with a distractor. In the present paper, we are particularly concerned with gender as a retrieval cue, as we use an experimental paradigm that manipulates gender agreement via reflexive-antecedent dependencies, allowing for the possibility of interference via a gender-matching distractor (see below for details). Under these circumstances, control dependencies would be expected to be susceptible to interference if they can use gender as a retrieval cue, while raising dependencies would be expected to be less susceptible, if their retrieval cues are purely structural. Finally, if nominal control and raising dependencies involve very different syntactic representations (e.g., if nominal control uses an empty infinitival subject, while raising does not, as suggested by the LTAG analysis, XTAG Research Group, 1998), then this could lead to different retrieval profiles for the two dependencies. We will postpone further discussion of this last point until the introduction to Experiment 4 below.

Our second hypothesis was that nominal control dependencies would be processed more slowly than raising dependencies. In order to examine this question, as well as retrieval interference, we used a *gender mismatch paradigm* (Sturt, 2003), combining raising or control dependencies with reflexive-antecedent gender agreement, as mentioned above. In this type of experiment, the matching between the reflexive and its antecedent is manipulated. For example, compare example (6) above, with the mis-matching variant in (7):

7. John seems to Mary Ø to be kind to herself.

In (7), the gender of the reflexive *herself* mismatches with the structurally appropriate antecedent *John*. Previous work, using eyetracking during reading, has shown that readers fixate for longer on a reflexive the when its gender mismatches that of its structurally licit antecedent (relative to matching controls) (see for example Sturt, 2003, inter alia).

In this paper, we refer to such processing difficulty as the *mismatch cost*, and we are particularly interested in the *onset* of the mismatch cost in the eye-movement record, in relation to the onset of the first fixation on the reflexive, as a measure of how quickly the grammatically appropriate antecedent is identified. In previous studies using eye-tracking, the mismatch cost for reflexive-antecedent dependencies has been observed very early in the eye-movement record. For example, Sturt (2003) reported that the first fixation on a reflexive with a gender mismatching antecedent was reliably shorter than when the antecedent matched in gender. Since the average fixation duration in reading is around 250 ms, this implies that the structurally appropriate antecedent must have been recognized within 250 ms after the reader first started fixating the reflexive.

In fact, there is some evidence to suggest that the *onset* of the mismatch cost may differ depending on the structure of the sentence that contains the reflexive. For example, in a series of eye-tracking experiments, Cunnings and Sturt (2014) used the gender mismatch paradigm to examine the resolution of reflexive pronouns sentences like (8a,b):

8a. He heard that the soldier had positioned himself/herself in the middle of the mess hall.
8b. He heard that the soldier had a picture of himself/herself in the middle of the mess hall.

The design included reflexives that either matched (*himself*) or mismatched (*herself*) the stereotypical gender of the antecedent (*the soldier*). In separate experiments, Cunnings and Sturt (2014) found evidence of a mismatch cost for both (8a) and (8b)—in both cases, readers began to slow down after they had initially fixated a mismatching reflexive (relative to a matching one). However, the onset of this mismatch cost appeared earlier in (8a) (where the reflexive and its antecedent *the soldier* are co-arguments of the same verb *positioned*), relative to (8b) (where the reflexive is embedded in a picture noun phrase, and is thus not a direct co-argument of the antecedent)^5^. This difference in the onset of the mismatch cost may indicate that the speed of dependency formation for reflexives is affected by the structure of the sentence—for example, it may be that initial retrieval processes consider co-arguments as potential antecedents, leading to an earlier formation of the dependency in (8a), and thus an earlier appearance of the mismatch cost.

The present research aims to follow up on these results by examining whether the onset of the mismatch cost for a reflexive is also affected by whether its antecedent is accessed via a raising or a nominal control dependency. There are several reasons why this may be the case. As mentioned above, the raising dependency can be resolved using purely structural information, while the control dependency requires a more complex evaluation of the control nominal's argument structure^6^. A second reason is related to the possibility that nominal control and raising may involve different syntactic representations. For example, if raising does not involve an empty infinitival subject, as suggested by the LTAG view XTAG Research Group (1998), then the dependency between a reflexive and its antecedent in an example like (6) is direct. This would contrast with nominal control, where the dependency would be assumed to be mediated by an empty subject. It may therefore be plausible to assume that a direct dependency might be processed more quickly than an indirect one, leading to an earlier onset of the mismatch cost for raising, relative to control.

In the remainder of this paper, we report four experiments that were designed to examine the formation of raising and nominal control dependencies. Experiment 1 establishes a baseline by examining reflexive-antecedent dependencies that are not mediated by raising or control. Experiment 2 directly compares raising and nominal control dependencies, without distractors, thus allowing us to test for differences in the onset of the mismatch cost. Then, in Experiments 3 and 4, we include distractors, focusing on nominal control (Experiment 3) and finally raising (Experiment 4).

We believe that it is important to consider a wide range of dependency types in our search to understand memory access and dependency formation in sentence comprehension. Raising and control dependencies offer a potentially interesting domain of enquiry, because they differ in theoretically relevant ways, while sharing considerable surface similarity. We also believe that it is important to consider not only simple direct dependencies between overt linguistic elements within a sentence, but also indirect dependencies, such as the reflexive-antecedent dependencies that are mediated by raising or control, which we examine here. We hope that the four experiments that we report below add new data points that will increase our understanding of the factors that affect retrieval interference, and will also provide a first step toward gaining a picture of retrieval in indirect dependencies.

## 2. Experiment 1

In Experiment 1, we establish a baseline by examining the processing of a direct dependency between a reflexive and its antecedent, without incorporating raising or control dependencies. In all other respects, the sentences are very similar to those used in the other experiments.

### 2.1. Materials and methods

#### 2.1.1. Participants

Thirty-two participants from the University of Edinburgh community were paid to participate in the experiment. All were native speakers of English, with normal or corrected-to-normal vision, and none reported any reading disability. All of the participants in the four experiments reported in this paper gave informed consent to take part. The research protocol was approved by the Psychology Research Ethics Committee, of the University of Edinburgh.

#### 2.1.2. Stimuli

The stimuli of Experiment 1 were similar to (9)^7^:

9a. **Accessible-match Inaccessible match:**
  John didn't trust Tom but was kind to himself appropriately and very sincerely.
9b. **Accessible-match Inaccessible mismatch:**
  John didn't trust Amy but was kind to himself appropriately and very sincerely.
9c. **Accessible-mismatch Inaccessible match:**
  Mary didn't trust Tom but was kind to himself appropriately and very sincerely.
9d. **Accessible-mismatch Inaccessible mismatch:**
  Mary didn't trust Amy but was kind to himself appropriately and very sincerely.

Given this design, the main effect of *accessible antecedent* matching can be used to gauge the time at which the parser first becomes sensitive to the gender matching between the reflexive and its grammatically correct antecedent, and can thus, given the assumptions above, be used as a measure of how quickly the structurally appropriate antecedent is identified. For example, if this effect is initially found in first fixation duration it would suggest that the antecedent is identified very early (see the Section 2.2 below for details of the eye-movement measures). Moreover, the effect of *inaccessible antecedent* (or its interaction with *accessible antecedent*) is informative about any effect of interference. For example, if the mismatch cost for the accessible antecedent is reduced where the inaccessible antecedent matches (11d) relative to when it does not (11c), this could be indicative of a *facilitatory interference* effect. Alternatively, if we find evidence for extra processing difficulty when both potential antecedents match the reflexive (11a) relative to when only the accessible antecedent matches (11b), then this could be interpreted as *inhibitory interference*. Given the experimental design, either of these two patterns, or their combination, would result in an interaction between the two experimental factors. Specifically, *facilitatory interference*, on its own, would result in a difference between the two accessible mismatch conditions (i.e., a penalty for inaccessible mismatch relative to inaccessible match), with no difference among the accessible match conditions. *inhibitory interference*, on its own, would result in a difference between the two accessible match conditions (i.e., a penalty for inaccessible match relative to inaccessible mismatch), with no difference among the accessible mismatch conditions. Finally, a combination of these two interference profiles would result in a cross-over pattern of means.

#### 2.1.3. Procedure

The experiment was carried out using an SR Research Eyelink 1000 eye-tracker, with a sampling rate of 1000 Hz. The tracker was used in tower mode. Only the right eye was tracked, although viewing was binocular. The eye-tracker was calibrated at the start of each participant's session, with recalibration being carried out as necessary through the experiment. At the start of each trial, a black box appeared at the left of the screen, in the position of the first character of text. When a stable fixation was detected in this position, the box disappeared, and the text appeared. The stimuli were presented in black on a white background, using Times Roman 16 point. The stimuli were presented in either one or two lines of text. In all cases, the critical reflexive was always placed at least two words before the end of a line.

The stimuli were combined with 102 filler sentences of varied sentence types. Thirty-six of the fillers were from an unrelated experiment on the processing of emotion words. A comprehension question followed around two thirds of all stimuli, including all of the experimental items (as an example, the question for (9) was “Was the kindness appropriate?”). The participant had to answer the question by pressing a button to select one out of two displayed answers. The stimuli were distributed into four lists, using latin square counterbalancing.

### 2.2. Data analysis

The sentences were divided into regions for the purpose of analysis. Here, we will report data for the following regions:

- **pre-critical region:** kind to
- **critical region (reflexive):** himself
- **spillover:** appropriately and
- **final:** very sincerely

Eye-fixation data were screened and manually corrected for vertical drift. Fixations of less than 80 ms were incorporated into larger fixations within a distance of one character, and then we deleted any remaining fixations of less than 80 ms, as well as any over 1200 ms.

We will report data for five eye-movement measures. First fixation is the duration of the first fixation in a region, from the time the region is first entered from the left, until a subsequent fixation is made. First pass reading time is the sum of fixation durations within the region, from the time the region is first entered from the left, until the region is exited, either to the left or right. Go-past is the sum of fixation durations from the time the region is first entered from the left until it is exited to the right (including any fixations made to the left of the region). Total time is the summed duration of all fixations on the region. In the above measures, for any given trial, if the measure returned no data (e.g., if there were no fixations on the region), the trial was treated as a missing value in the analysis. Finally, Second Pass reading time is the summed duration of all re-fixations on the region, after it has already been fixated for the first time. As is customary, for Second Pass reading time, trials that do not include a relevant fixation are included in the analysis as zero millisecond data points. Note that the first fixation measure is most meaningfully applied to single-word regions, which can be assumed to be processable within a single fixation. Thus, we report first fixation durations only for the critical reflexive region.

The results for all eye-movement measures were submitted to 2 × 2 Analyses of variance, aggregating by subject (*F*_1_) and by item (*F*_2_). The factors in the analysis were Accessible antecedent matching (match vs. mismatch) and Inaccessible antecedent matching (match vs. mismatch), both of which were within item and within participant.

### 2.3. Results

Two items were excluded from analysis because of typographical errors. Therefore, the item analysis is based on 38 items, with a corresponding reduction in the degrees of freedom for the *F*_2_ analysis. Means for Experiment 1 are presented in Table 1, and statistical results are presented in Table 2.

**Table 1:** **Means (and Standard Errors) for Experiment 1**.

**Region**		**Pre-critical**	**Critical**	**Spillover**	**Final**
**Accessible**	**Inaccessible**				
**FIRST FIXATION**					
Match	Match	–	239 (9)	–	–
Match	Mismatch	–	245 (9)	–	–
Mismatch	Match	–	251 (8)	–	–
Mismatch	Mismatch	–	262 (10)	–	–
**FIRST PASS**					
Match	Match	308 (16)	256 (12)	517 (35)	397 (24)
Match	Mismatch	329 (18)	271 (10)	500 (29)	465 (29)
Mismatch	Match	337 (19)	286 (14)	488 (32)	412 (27)
Mismatch	Mismatch	334 (21)	291 (13)	508 (31)	425 (30)
**GO-PAST**					
Match	Match	396 (26)	343 (22)	722 (64)	1482 (119)
Match	Mismatch	388 (21)	357 (19)	707 (54)	1463 (123)
Mismatch	Match	438 (42)	367 (18)	816 (61)	1667 (159)
Mismatch	Mismatch	403 (25)	378 (26)	914 (66)	1628 (167)
**SECOND PASS**					
Match	Match	224 (29)	153 (21)	298 (32)	126 (17)
Match	Mismatch	190 (24)	143 (19)	292 (30)	133 (20)
Mismatch	Match	244 (34)	176 (19)	360 (42)	139 (22)
Mismatch	Mismatch	223 (22)	179 (19)	392 (40)	159 (23)
**TOTAL TIME**					
Match	Match	525 (34)	404 (25)	813 (51)	533 (33)
Match	Mismatch	514 (32)	406 (23)	795 (47)	609 (38)
Mismatch	Match	565 (45)	447 (27)	845 (62)	560 (38)
Mismatch	Mismatch	548 (39)	457 (24)	898 (59)	600 (47)

**Table 2:** Anova results for Experiment 1 (^+^*p* < 0.1; ^*^p < 0.05; ^**^p < 0.01; ^***^p < 0.001).

**Region**	**Pre-critical**		**Critical**		**Spillover**		**Final**	
	***F*_(1, 31)_**	***F*_2(1, 37)_**	***F*_(1, 31)_**	***F*_2(1, 37)_**	***F*_(1, 31)_**	***F*_2(1, 37)_**	***F*_(1, 31)_**	***F*_2(1, 37)_**
**FIRST FIXATION**								
Accessible	–	–	5.51^*^	5.54^*^	–	–	–	–
Inaccessible	–	–	2.21	1.57	–	–	–	–
Acc ^*^ Inacc	–	–	<1	<1	–	–	–	–
**FIRST PASS**								
Accessible	4.37^*^	1.34	6.98^*^	7.20^*^	<1	<1	<1	<1
Inaccessible	<1	<1	1.85	2.72	<1	<1	6.18^*^	3.84^+^
Acc ^*^ Inacc	1.32	1.39	<1	<1	1.23	<1	1.46	5.81^*^
**GO-PAST**								
Accessible	1.87	2.20	1.45	2.84	17.98^***^	10.36^**^	3.18^+^	6.77^*^
Inaccessible	1.47	1.45	<1	<1	1.50	<1	<1	<1
Acc^*^Inacc	<1	<1	<1	<1	2.10	2.26	<1	<1
**SECOND PASS**								
Accessible	1.26	3.74^+^	5.15^*^	7.25^*^	12.84^**^	10.65^**^	<1	1.82
Inaccessible	3.04^+^	1.77	<1	<1	<1	<1	<1	1.19
Acc ^*^ Inacc	<1	<1	<1	<1	<1	<1	<1	<1
**TOTAL TIME**								
Accessible	2.06	4.08^+^	10.40^**^	14.88^***^	6.16^*^	8.43^**^	<1	<1
Inaccessible	<1	<1	<1	<1	<1	<1	7.12^*^	4.04^+^
Acc ^*^ Inacc	<1	<1	<1	<1	2.21	2.30	<1	2.07

As in previous work (e.g., Sturt, 2003), there was very early evidence for a mismatch cost for the accessible antecedent; the effect appeared in the first fixation duration on the critical reflexive (the earliest measurable point, given the eye-tracking methodology), and this was mirrored in first pass times in the same region. However, this early effect did not interact with the matching of the inaccessible antecedent. The inaccessible antecedent had a marginal effect on fixation times in the final region in Total Time and First Pass. The pattern was for the inaccessible mismatch condition to lead to longer reading times than the corresponding match condition. In First Pass, this effect in the final region interacted with the accessible antecedent, but only in the analysis by item—the reading time penalty for the inaccessible mismatch condition (relative to inaccessible match) was greater when the reflexive matched the accessible antecedent (465 vs. 397 ms; a relative cost of 68 ms; both *F*'s > 6, both *p*'s < 0.02) than when it did not (425 vs. 412 ms; a relative cost of 13 ms; both *F*'s < 1).

### 2.4. Discussion

This experiment sets a baseline using direct reflexive-antecedent dependencies, for the following experiments, where the reflexive-antecedent dependency is mediated by raising and control. We find that an early main effect of accessible antecedent on the critical reflexive, indicating an early onset of the mismatch cost. There was little evidence of either inhibitory interference or facilitatory interference, at least in the early measures. Later effects suggest a difficulty for *mismatching*, relative to *matching* inaccessible antecedents. This pattern may possibly be interpreted in terms of facilitatory interference. However, this interpretation is not straightforward, as the effect of inaccessible antecedent appeared as a main effect rather than the interaction predicted by current memory models. In fact, the marginal interaction in First Pass in the final region shows, if anything, that the facilitatory effect was larger for the *grammatical* sentences than the ungrammatical sentences, which is not the pattern that is expected for facilitatory interference. In addition, we note that First Pass reading times are often hard to interpret in the final region of a sentence, due to the possibility of relatively short initial fixations preceding regressions out of the region (see Sturt, 2007; Sturt et al., 2010).

## 3. Experiment 2

Experiment 2 used reflexive-antecedent dependencies that are mediated by raising or nominal control, depending on condition, in simple sentences that do not contain distractor noun phrases. This allows us to determine whether there are any baseline differences in the time-course of processing of raising-mediated and control-mediated dependencies, over and above those that may be explained in terms of interference effects. If the dependencies are formed more quickly when they are mediated by raising than when they are mediated by control, then we would expect the onset of the mismatch cost to appear earlier in the eye-movement record in raising than in control.

## 4. Materials and methods

### 4.1. Participants

Thirty-two participants from the University of Edinburgh community were paid to participate in the experiment. All were native speakers of English, with normal or corrected-to-normal vision, and none reported any reading disability.

### 4.2. Stimuli

There were 40 stimuli, which were similar to those given in (10):

10a. **Control Match:**
  I was surprised at John's agreement to be kind to himself appropriately and very sincerely.
10b. **Control Mismatch:**
  I was surprised at John's agreement to be kind to herself appropriately and verysincerely.
10c. **Raising Match:**
  It was surprising that John seemed to be kind to himself appropriately and very sincerely.
10d. **Raising Mismatch:**
  It was surprising that John seemed to be kind to herself appropriately and very sincerely.

The design manipulated sentence type (Raising vs. Control), and gender matching (Match vs. Mismatch).

As we mentioned in the introduction, we assume that the raising and nominal control dependencies are initially formed around the point where *to be kind* is received in the input, and that there is a second retrieval event that is triggered by the reflexive, which is also mediated by control (10a,b) or raising (10c,d). It is this second retrieval event that we are measuring in this experiment, using the gender-mismatch paradigm. It is important to recognize that this second retrieval event involves two dependencies, (a) a reflexive-antecedent dependency (between *himself* and its direct antecedent, the empty subject of the infinitival clause), and (b) a raising or control dependency (between the empty subject and *John*). The logic of the design is that, as the relevant aspects of the reflexive-antecedent dependency are essentially identical between the raising and control conditions, any differences that we might find in the onset of the mismatch cost must be due to processing differences related to raising or control.

### 4.3. Procedure

The sentences were divided into regions for the purpose of analysis as shown below.

- **pre-critical region:** kind to
- **critical region:** himself
- **spillover:** appropriately and
- **final:** very sincerely

The pre-critical region consisted of the two words immediately preceding the critical reflexive. The spillover region consisted of the two words immediately following the reflexive. The final region consisted of the last two words of the sentence.

### 4.4. Results

The means are given in Table 3, and statistical results in Table 4.

**Table 3:** **Means (and Standard Errors) for Experiment 2**.

**Region**		**Pre-critical**	**Critical**	**Spillover**	**Final**
**FIRST FIXATION**					
Control	Match	–	251 (8)	–	–
Control	Mismatch	–	265 (9)	–	–
Raising	Match	–	245 (9)	–	–
Raising	Mismatch	–	262 (9)	–	–
**FIRST PASS**					
Control	Match	343 (19)	274 (10)	567 (43)	412 (31)
Control	Mismatch	348 (19)	296 (14)	547 (43)	454 (42)
Raising	Match	339 (19)	274 (12)	516 (39)	419 (29)
Raising	Mismatch	323 (16)	300 (12)	515 (40)	429 (27)
**GO-PAST**					
Control	Match	449 (26)	366 (26)	819 (73)	1914 (294)
Control	Mismatch	507 (35)	454 (44)	928 (79)	2006 (286)
Raising	Match	450 (32)	366 (23)	727 (64)	1767 (231)
Raising	Mismatch	447 (27)	462 (42)	1030 (85)	1848 (247)
**SECOND PASS**					
Control	Match	293 (50)	189 (30)	438 (78)	186 (28)
Control	Mismatch	315 (49)	245 (35)	496 (70)	198 (33)
Raising	Match	239 (33)	181 (30)	367 (44)	185 (32)
Raising	Mismatch	304 (45)	259 (37)	509 (55)	196 (30)
**TOTAL TIME**					
Control	Match	636 (60)	456 (33)	1006 (101)	602 (49)
Control	Mismatch	656 (54)	530 (39)	1043 (86)	659 (63)
Raising	Match	575 (47)	448 (32)	880 (67)	613 (50)
Raising	Mismatch	621 (52)	545 (41)	1023 (75)	633 (46)

**Table 4:** Anova results for Experiment 2 (^+^*p* < 0.1; ^*^p < 0.05; ^**^p < 0.01; ^***^p < 0.001).

**Region**	**Pre-critical**		**Critical**		**Spillover**		**Final**	
	***F*_1(1, 31)_**	***F*_2(1, 39)_**	***F*_1(1, 31)_**	***F*_2(1, 39)_**	***F*_1(1, 31)_**	***F*_2(1, 39)_**	***F*_1(1, 31)_**	***F*_2(1, 39)_**
**FIRST FIXATION**								
Structure	–	–	1.61	<1	–	–	–	–
Matching	–	–	8.29^**^	8.30^**^	–	–	–	–
Struc ^*^ Match	–	–	<1	<1	–	–	–	–
**FIRST PASS**								
Structure	1.97	2.29	<1	<1	4.47^*^	4.02^+^	<1	<1
Matching	<1	<1	13.39^**^	10.39^**^	<1	<1	2.32	2.20
Struc ^*^ Match	<1	<1	<1	<1	<1	<1	<1	<1
**GO-PAST**								
Structure	1.87	1.97	<1	<1	<1	<1	2.10	<1
Matching	1.79	1.36	8.65^**^	16.73^***^	13.93^**^	20.26^***^	1.44	1.36
Struc ^*^ Match	2.69	1.83	<1	<1	2.77	3.60^+^	<1	<1
**SECOND PASS**								
Structure	2.84	2.27	<1	<1	<1	<1	<1	<1
Matching	4.79^*^	8.75^**^	13.58^**^	7.68^**^	11.49^**^	14.21^**^	<1	<1
Struc ^*^ Match	<1	1.43	<1	<1	3.18^+^	1.69	<1	<1
**TOTAL TIME**								
Structure	6.04^*^	5.10^*^	<1	<1	2.63	4.51^*^	<1	<1
Matching	1.85	3.09^+^	18.16^***^	9.72^**^	8.36^**^	6.37^*^	2.85	1.37
Struc ^*^ Match	<1	<1	<1	<1	2.91^+^	2.29	<1	<1

As in Experiment 1, there was an early effect of matching, indicating a cost for the gender mis-matching items. This effect is present in all eye-movement measures on the critical reflexive, and persisted into the spill-over region. As this includes measures indicative of early processing, such as first-pass reading time, and first fixation, this suggests that the antecedent was identified equally quickly, whether the dependency was mediated by raising or control. In fact, the timing was in line with the co-argument reflexive-antecedent dependencies examined in Experiment 1. This early mismatch cost did not interact with structure. In addition, a main effect of structure type suggested that the control sentences were harder to read than the raising sentences (see Total Time, pre-critical region, and First Pass, spill-over region). However, this overall difference is not the focus of the current investigation.

### 4.5. Discussion

In this experiment, we investigated sentences where raising and control dependencies were combined with reflexive-antecedent dependencies. The main effect of matching appeared in both first fixation and first pass on the critical reflexive. This is the earliest detectable point given the eye-tracking methodology, and is in line with the timing of the accessible mismatch effect in the co-argument reflexive-antecedent dependencies examined in Experiment 1. As the effect was not modulated by sentence structure, there is no indication of any difference in the time-course of antecedent identification, whether the dependency was mediated by raising or nominal control dependencies. However, the study was carried out as a baseline, and did not include distractor noun phrases. Thus, although the study suggests no clear difference in time-course between dependency types, it does not rule out that the two dependency types may be differentially susceptible to interference. In Experiments 3 and 4, we address this issue, by including distractor antecedents in Nominal control (Experiment 3) and Raising (Experiment 4) sentences.

## 5. Experiment 3

Experiment 3 was designed to test the susceptibility of nominal control dependencies to interference.

### 5.1. Materials and methods

#### 5.1.1. Participants

Thirty-two new participants from the University of Edinburgh community were paid to participate in the experiment. All were native speakers of English, with normal or corrected-to-normal vision, and none reported any reading disability.

#### 5.1.2. Stimuli

There were forty experimental items similar to those in (11)^8^:

11a. **Accessible-match Inaccessible match:**
  John's agreement with Tom to be kind to himself was surprising to everyone.
11b. **Accessible-match Inaccessible mismatch:**
  John's agreement with Amy to be kind to himself was surprising to everyone.
11c. **Accessible-mismatch Inaccessible match:**
  Mary's agreement with Tom to be kind to himself was surprising to everyone.
11d. **Accessible-mismatch Inaccessible mismatch:**
  Mary's agreement with Amy to be kind to himself was surprising to everyone.

The items all used giver control nominals (exemplified by *agreement* in 11a–d; Culicover and Jackendoff, 2001), with the result that the accessible antecedent for the reflexive was always the genitive subject of the control nominal (e.g., *John's* in 11). The design orthogonally manipulated the gender matching of the reflexive with the accessible antecedent (e.g., *Mary* vs. *John*) and with the inaccessible antecedent (*Tom* vs. *Amy*).

#### 5.1.3. Procedure

All relevant aspects of the procedure were identical to Experiment 1.

We will report analyses based on the following regions:

- **pre-critical region:** kind to
- **critical region (reflexive):** himself
- **spillover:** was surprising
- **final:** to everyone.

### 5.2. Results

The means are given in Table 5, and statistical results in Table 6.

**Table 5:** **Means (and Standard Errors) for Experiment 3**.

**Region**		**Pre-critical**	**Critical**	**Spillover**	**Final**
**Accessible**	**Inaccessible**				
**FIRST FIXATION**					
Match	Match	–	238 (8)	–	–
Match	Mismatch	–	230 (8)	–	–
Mismatch	Match	–	245 (10)	–	–
Mismatch	Mismatch	–	239 (9)	–	–
**FIRST PASS**					
Match	Match	313 (19)	254 (9)	487 (26)	358 (40)
Match	Mismatch	322 (16)	257 (11)	462 (21)	357 (29)
Mismatch	Match	317 (17)	266 (12)	440 (22)	334 (29)
Mismatch	Mismatch	331 (20)	260 (13)	465 (26)	331 (31)
**GO-PAST**					
Match	Match	415 (29)	307 (14)	794 (76)	1138 (112)
Match	Mismatch	406 (22)	340 (25)	768 (58)	1165 (107)
Mismatch	Match	399 (20)	386 (36)	797 (83)	1198 (147)
Mismatch	Mismatch	414 (27)	388 (39)	948 (71)	1305 (158)
**SECOND PASS**					
Match	Match	190 (26)	97 (15)	180 (25)	60 (12)
Match	Mismatch	202 (27)	114 (18)	217 (26)	64 (12)
Mismatch	Match	205 (31)	134 (23)	233 (37)	58 (15)
Mismatch	Mismatch	239 (34)	174 (23)	272 (31)	61 (11)
**TOTAL TIME**					
Match	Match	500 (31)	343 (19)	664 (43)	470 (55)
Match	Mismatch	514 (32)	364 (21)	679 (36)	443 (40)
Mismatch	Match	517 (34)	394 (27)	673 (50)	407 (34)
Mismatch	Mismatch	565 (39)	434 (29)	733 (45)	414 (42)

**Table 6:** Anova results for Experiment 3 (^+^*p* < 0.1; ^*^p < 0.05; ^**^p < 0.01; ^***^p < 0.001).

**Region**	**Pre-critical**		**Critical**		**Spillover**		**Final**	
	***F*_1(1, 31)_**	***F*_2(1, 39)_**	***F*_1(1, 31)_**	***F*_2(1, 39)_**	***F*_1(1, 31)_**	***F*_2(1, 39)_**	***F*_1(1, 31)_**	***F*_2(1, 39)_**
**FIRST FIXATION**								
Accessible	–	–	1.61	3.21^+^	–	–	–	–
Inaccessible	–	–	1.95	<1	–	–	–	–
Acc ^*^ Inacc	–	–	<1	<1	–	–	–	–
**FIRST PASS**								
Accessible	<1	<1	<1	1.18	5.01^*^	1.94	1.67	1.00
Inaccessible	1.57	2.51	<1	<1	<1	<1	<1	<1
Acc ^*^ Inacc	<1	<1	<1	<1	3.19^+^	3.57^+^	<1	<1
**GO-PAST**								
Accessible	<1	<1	5.38^*^	7.18^*^	3.43^+^	4.16^*^	1.17	1.21
Inaccessible	<1	<1	<1	1.88	3.01^+^	1.29	<1	<1
Acc ^*^ Inacc	<1	<1	<1	<1	3.17^+^	3.65^+^	<1	<1
**SECOND PASS**								
Accessible	2.53	3.03^+^	14.81^**^	16.63^***^	11.20^**^	7.42^*^	<1	<1
Inaccessible	1.44	1.07	5.38^*^	2.64	7.43^*^	1.85	<1	<1
Acc ^*^ Inacc	<1	<1	1.02	<1	<1	<1	<1	<1
**TOTAL TIME**								
Accessible	4.14^+^	4.25^*^	17.78^***^	27.39^***^	3.13^+^	1.44	2.76	<1
Inaccessible	3.29^+^	1.40	3.87^+^	2.15	3.91^+^	<1	<1	<1
Acc ^*^ Inacc	<1	<1	<1	<1	<1	<1	<1	<1

The results show evidence of a mismatch cost for the accessible antecedent in go-past, total time and second pass in the critical reflexive region. Go-past and Total times on this region were not modulated by any interactions with inaccessible antecedent matching. There was some marginal evidence that reading was affected by the inaccessible antecedent, in measures of later processing. The effect of inaccessible matching was significant (in the subjects analysis only) in second pass in both the critical and spill-over regions; as in Experiment 1, the tendency was for inaccessible mismatch conditions to be read more slowly than inaccessible match conditions.

There was a marginal interaction between accessible and inaccessible gender matching in go-past and first-pass reading time in the spill-over region. This interaction was examined using pairwise comparisons, to test the simple effect of inaccessible antecedent, within (a) the accessible match conditions, and (b) the accessible mismatch conditions. For first-pass reading times, neither of these pairwise comparisons was reliable (all *p*'s > 0.1). However, for Go-Past time, pairwise comparison (b) (i.e., within the accessible mismatch conditions) showed significantly faster reading times for the inaccessible match (797 ms) relative to inaccessible mismatch (948 ms) [*F*_1(1, 31)_ = 5.72, *p* < 0.05; *F*_2(1, 39)_ = 4.11, *p* < 0.05], while comparison (a) (i.e., within the accessible match conditions) showed a non-significant difference in the opposite direction (794 ms vs. 768 ms) [both *p*'s < 1].

### 5.3. Discussion

The first appearance of the mismatch cost for the accessible antecedent was in the Go-past measure on the critical reflexive. This shows that the ungrammatical dependency in the accessible mismatch conditions disrupted processing fairly quickly—soon after the participants initially fixated the reflexive, and before they moved on to fixate subsequent words.

The experiment did not show strong interference effects, but we believe that the results for Go-past in the spill-over region are highly suggestive, at the very least. Despite the fact that the interaction was marginal, the results of the pairwise comparisons are as predicted for facilitatory interference, since the cost for the accessible mismatch was significantly reduced when the inaccessible antecedent matched the gender of the reflexive, relative to when it did not.

## 6. Experiment 4

Experiment 1 showed no evidence that could be straightforwardly interpreted in terms of interference, for direct reflexive-antecedent dependencies that were not mediated by raising or control. Experiment 3 showed some marginal evidence for facilitatory interference, in the resolution of reflexive-antecedent dependencies that were mediated by nominal control. In Experiment 4, we examine reflexive-antecedent dependencies that are mediated by raising, using a design that is analogous to that of Experiment 3.

### 6.1. Materials and methods

#### 6.1.1. Participants

Thirty-two new participants from the University of Edinburgh community were paid to participate in the experiment. All were native speakers of English, with normal or corrected-to-normal vision, and none reported any reading disability.

#### 6.1.2. Stimuli

There were 40 stimuli, which were similar to those in (12)

12a. **Accessible-match Inaccessible match:**
  John seemed to Tom to be kind to himself appropriately and very sincerely.
12b. **Accessible-match Inaccessiblex mismatch:**
  John seemed to Amy to be kind to himself appropriately and very sincerely.
12c. **Accessible-mismatch Inaccessible match:**
  Mary seemed to Tom to be kind to himself appropriately and very sincerely.
12d. **Accessible-mismatch Inaccessible mismatch:**
  Mary seemed to Amy to be kind to himself appropriately and very sincerely.

The items used a raising construction incorporating an experiencer argument (e.g., *to Amy*). The accessible antecedent for the reflexive was always the subject of the main clause (e.g., *Mary*), while the experiencer argument was always an inaccessible antecedent. The design orthogonally manipulated the gender matching of accessible and inaccessible antecedents.

Recall from the introduction of this paper that we expected raising-mediated dependencies to be less susceptible to interference than the control-mediated dependencies that we examined in Experiment 3. The introduction lists some reasons for this expectation, such as potential differences in access mechanisms, retrieval cues, or syntactic representation. Here, we will briefly elaborate on how differences in syntactic representation may lead to different retrieval profiles, using Lexicalized Tree Adjoining Grammar (LTAG) as an example grammatical framework. In LTAG, the matrix subject in (12) (e.g., *John*), would be assumed to occupy the subject position of a predicative elementary tree^9^, projected by *kind*, without this relationship being mediated by an empty subject position in the infinitival clause (see XTAG Research Group, 1998, p.106–107). In contrast, in the nominal control stimuli (see 11 in Experiment 3), *John's* would be assumed to occupy the specifier position of *agreement*, while the infinitival clause would have an empty subject, occupied by the empty element PRO (see XTAG Research Group, 1998, p.97–101, for an analysis of verbal control)^10^. Thus, according to the LTAG proposal, *John* is effectively a co-argument of *himself* in the raising sentences, but is not a direct co-argument of *himself* in the nominal control sentences. Accordingly, this approach would predict that the interference profile for raising-mediated dependencies would pattern like the co-argument dependencies examined in Experiment 1, rather than like the control-mediated dependencies examined in Experiment 2.

#### 6.1.3. Procedure

As the experiment was based on Experiment 3, the regions were defined identically:

- **pre-critical region:** kind to
- **critical region (reflexive):** himself
- **spillover:** appropriately and
- **final:** very sincerely

### 6.2. Results

The means are given in Table 7, and statistical results in Table 8.

**Table 7:** **Means (and Standard Errors) for Experiment 4**.

**Region**		**Pre-critical**	**Critical**	**Spillover**	**Final**
**Accessible**	**Inaccessible**				
**FIRST FIXATION**					
Match	Match	–	246 (8)	–	–
Match	Mismatch	–	255 (8)	–	–
Mismatch	Match	–	257 (8)	–	–
Mismatch	Mismatch	–	263 (8)	–	–
**FIRST PASS**					
Match	Match	313 (18)	277 (9)	510 (29)	442 (28)
Match	Mismatch	310 (15)	289 (9)	518 (38)	458 (32)
Mismatch	Match	318 (19)	293 (11)	534 (31)	422 (24)
Mismatch	Mismatch	313 (14)	298 (11)	498 (26)	412 (24)
**GO-PAST**					
Match	Match	407 (25)	333 (15)	775 (61)	1741 (177)
Match	Mismatch	404 (21)	379 (24)	782 (60)	1518 (152)
Mismatch	Match	400 (23)	427 (49)	882 (57)	1832 (211)
Mismatch	Mismatch	442 (34)	461 (35)	960 (67)	1955 (212)
**SECOND PASS**					
Match	Match	220 (27)	153 (23)	359 (37)	206 (29)
Match	Mismatch	225 (26)	149 (17)	337 (36)	150 (21)
Mismatch	Match	294 (37)	215 (21)	398 (44)	208 (33)
Mismatch	Mismatch	323 (41)	266 (31)	519 (62)	221 (28)
**TOTAL TIME**					
Match	Match	533 (32)	426 (28)	871 (52)	672 (47)
Match	Mismatch	533 (28)	431 (18)	857 (57)	618 (44)
Mismatch	Match	607 (41)	499 (25)	932 (55)	645 (41)
Mismatch	Mismatch	626 (45)	541 (31)	1016 (73)	647 (40)

**Table 8:** Anova results for Experiment 4 (^+^*p* < 0.1; ^*^p < 0.05; ^**^p < 0.01; ^***^p < 0.001).

**Region**	**Pre-critical**		**Critical**		**Spillover**		**Final**	
	***F*_1(1, 31)_**	***F*_2(1, 39)_**	***F*_1(1, 31)_**	***F*_2(1, 39)_**	***F*_1(1, 31)_**	***F*_2(1, 39)_**	***F*_1(1, 31)_**	***F*_2(1, 39)_**
**FIRST FIXATION**								
Accessible	–	–	2.37	1.18	–	–	–	–
Inaccessible	–	–	2.55	<1	–	–	–	–
Acc ^*^ Inacc	–	–	<1	<1	–	–	–	–
**FIRST PASS**								
Accessible	<1	<1	1.98	1.66	<1	<1	3.84^+^	5.69^*^
Inaccessible	<1	<1	1.30	<1	<1	<1	<1	<1
Acc ^*^ Inacc	<1	<1	<1	<1	<1	<1	<1	<1
**GO-PAST**								
Accessible	<1	<1	7.43^*^	11.03^**^	15.90^***^	6.28^*^	7.09^*^	18.75^***^
Inaccessible	1.42	<1	2.49	2.79	1.41	<1	<1	<1
Acc ^*^ Inacc	1.75	1.28	<1	<1	<1	<1	7.42^*^	2.35
**SECOND PASS**								
Accessible	17.76^***^	26.27^***^	37.35^***^	31.87^***^	12.81^**^	14.47^***^	2.89^+^	5.22^*^
Inaccessible	<1	1.03	1.61	2.59	3.79^+^	3.18^+^	1.83	1.03
Acc ^*^ Inacc	<1	<1	5.63^*^	4.93^*^	6.17^*^	5.94^*^	3.00^+^	4.98^*^
**TOTAL Time**								
Accessible	8.82^**^	19.42^***^	25.03^***^	25.92^***^	12.08^**^	10.26^**^	<1	<1
Inaccessible	<1	<1	1.37	1.34	1.59	1.84	<1	<1
Acc ^*^ Inacc	<1	<1	1.46	<1	1.86	1.72	1.09	1.07

As with Experiment 3, the first evidence of a mismatch cost for the accessible antecedent is in the go-past measure on the critical reflexive region, with a main effect of accessible matching. This main effect persists until the final region, and is found (in the critical and spill-over regions) also in the Total Time and Second Pass measures.

Second pass reading time shows a significant interaction between accessible and inaccessible matching in both the critical and spill-over regions. Pair-wise comparisons on both of these regions show a pattern consistent with facilitatory interference: there was a reliable difference between the two accessible mismatch conditions, with longer second pass times when the inaccessible antecedent also mismatches the reflexive than when it does not {critical region: 266 ms vs. 215 ms [*F*_1(1, 31)_ = 4.10, *p* = 0.052; *F*_2(1, 39)_ = 5.66, *p* < 0.05]; spill-over region: 519 ms vs. 398 ms [*F*_1(1, 31)_ = 6.92, *p* < 0.05; *F*_2(1, 39)_ = 7.68, *p* < 0.01]}. In contrast, the difference between the two accessible match conditions was in the other direction, but much smaller, and non-significant (critical region: 149 ms vs. 153 ms; spill-over region: 337 ms vs. 359 ms; all *F*'s < 1).

On the final region, there were marginal interactions in both Second pass and Go-past (significant only by subjects for Go-past, and only by items for Second-pass). Pairwise comparisons revealed patterns of significance that were suggestive of inhibitory interference. Among the accessible match conditions, reading times were longer when the inaccessible antecedent also matched the reflexive, than when it did not {Go-past: 1741 ms vs. 1518 ms; [*F*_1(1, 31)_ = 4.63, *p* < 0.05; *F*_2_(1, 39) = 1.60, *p* = 0.21]; Second pass: 206 ms vs. 150 ms; [*F*_1(1, 31)_ = 4.32, *p* < 0.05; *F*_2_(1, 39) = 5.54, *p* < 0.05]}. Among the accessible mismatch conditions, the difference was in the opposite direction, but was not reliable (Go-past: 1832 ms vs. 1955 ms; both *F*'s <1.2, both *p*'s > 0.3; Second pass: 208 ms vs. 221 ms; both *F*'s < 1).

### 6.3. Discussion

As in Experiment 3, the first indication of a mismatch cost was the main effect of accessible matching in the critical reflexive region. There was also some clear evidence of facilitatory interference, in second-pass reading times in the critical and spill-over regions. Thus, the interference profile for this raising-mediated dependency resembled that of the control-mediated dependencies in Experiment 3, and differed from the the co-argument dependencies examined in Experiment 1, where no strong evidence of interference was found. Thus there is no evidence for the hypothesis that raising-mediated dependencies should show reduced interference effects relative to control-mediated dependencies, based on differences in the access mechanism, retrieval cues, or syntactic representation.

Unlike any of the previous experiments, there was also some evidence of inhibitory interference. However, this result is hard to interpret, as it comes from second-pass and go-past measures on the final region, and could thus be contaminated by wrap-up effects, or preparations for the comprehension question. Here, second pass time is based on the fixations that are made when the final region is re-fixated, following any initial regressions back to earlier points in the sentence, and before the button is pressed to indicate the end of the trial. Go-past time on this region also includes these fixations. Thus, if inhibitory interference is indeed present, it occurred very late in the trial, probably during sentence-final wrap-up.

## 7. General discussion

The above experiments were designed to examine the interference profile, and speed of dependency formation, for raising and nominal control dependencies. We began with the hypothesis that nominal control dependencies would be more subject to interference, and processed more slowly, than raising dependencies. This prediction was not confirmed overall. In the following, we will discuss the issues of time-course and interference in turn.

Experiment 1 established a baseline using reflexive-antecedent dependencies without the involvement of raising or control, and it replicated previous work in showing that gender mismatching between a reflexive and its accessible antecedent can slow down processing as early as the first fixation on the reflexive. Experiment 2 further established that, in the absence of inaccessible distractor antecedents, dependencies that were mediated by raising and nominal control elicited an equally early onset of the gender mismatch difficulty. Experiments 3 (control) and 4 (raising) included inaccessible distractor antecedents. These experiments showed the accessible mismatching cost on the critical reflexive in go-past, as well as in Total Time and Second pass, but, unlike in Experiments 1 and 2, not in first-fixation or first-pass.

Although we need to be cautious in interpreting between-experiment differences among first-pass measures, the control-mediated dependencies did not show an earlier onset for the mismatch cost than raising-mediated dependencies. Instead, the overall pattern of results is consistent with a slightly delayed onset of the mismatch cost for both the raising and control dependencies in Experiments 3 and 4 (go-past on the critical reflexive), relative to the co-argument reflexive-coargument dependencies tested in Experiment 1 (first-fixation and first pass on the critical reflexive). This delayed onset does not appear to be due to the involvement of raising or control dependencies *per se*, as Experiment 2, which used these dependencies (but without distractor phrases), showed an onset of mismatch difficulty in first-fixation, as early as that of Experiment 1. Rather, if anything, the delayed onset appears to be due to the presence of potentially interfering distractor phrases (whatever their gender marking), in conjunction with the use of raising and control dependencies. This should be interpreted as a preliminary finding, pending further investigation using more complex within-participant designs that have sufficient power to allow the statistical detection of potentially small differences in the onset of the mismatch cost. Such studies could also be supplemented by studies that allow a more direct measure of processing speed (e.g., Speed Accuracy Tradeoff; McElree et al., 2003).

Turning now to the discussion of interference, the results did not support the idea that dependencies mediated by nominal control would be more susceptible to interference than raising dependencies. On the one hand, assuming that the marginal interaction effect for Experiment 3 (control) reflects genuine interference, it may be the case that interference occurs earlier where the dependency is mediated by nominal control, compared with when it is mediated by raising. This is because the interference effect for Experiment 3 occurred shortly after readers had progressed forwards from the critical reflexive (i.e., in Go-past on the spill-over region), while in Experiment 4 (raising), the same region showed the effect only in second-pass. On the other hand, the interference effect seems to be stronger in Experiment 4 (raising) than in Experiment 3 (control). That is, in Experiment 3, the interaction between accessible and inaccessible gender matching was (marginally) significant only in go-past and first-pass reading time in the spill-over region, while in Experiment 4 it was fully significant in second-pass on the critical and spill-over regions (and marginal in two measures on the final region of the sentence). Thus, overall patterns of results do not support the hypothesis that the involvement of lexically-driven dependencies (control) leads to more interference than that of structurally-driven dependencies (raising), or that the access mechanism differs due to different retrieval cues or syntactic representations.

Both Experiment 3 (control) and Experiment 4 (raising) showed the profile expected for facilitatory interference. The pattern was such that when the reflexive did not match the gender of its structurally licit antecedent, the processing cost was reduced if there was an intervening distractor that matched the reflexive, relative to when the distractor did not match. The fact that interference was facilitatory, rather than inhibitory, accords with previous studies on subject-verb agreement (e.g., Wagers et al., 2009) and negative polarity licensing (e.g., Vasishth et al., 2008; Xiang et al., 2009), where interference was found only among ungrammatical (or otherwise degraded) conditions. Thus, like those earlier studies, our results do not tell us whether interference also affects grammatical, non-degraded dependencies. Moreover, our interference effect was found in measures that reflect fixation behavior after the reader has already progressed forwards from the critical reflexive, and thus, after the point where the mismatching of the accessible antecedent had started to cause a slow-down in reading. Because of this, we believe that the retrieval interference for these dependencies occurred, not during the initial retrieval of the antecedent, but during the repair process, possibly reflecting a *re-retrieval*, while readers searched for an acceptable interpretation of the ungrammatical sentences in the accessible mismatch conditions. In fact, the pattern of results can be summarized by saying that, while the *onset* of the accessible mismatch cost was unaffected by the gender of the distractor, the *duration* of this processing difficulty was affected by the distractor—i.e., the duration was shorter when the distractor matched the reflexive's gender.

Recall that the stimuli of Experiments 3 and 4 used dependencies that involved both reflexive-antecedent dependencies and control (or raising) dependencies. However, Experiment 1 used co-argument reflexive-antecedent dependencies with superficially very similar materials, and it showed no reliable evidence that could be straightforwardly interpreted in terms of the facilitatory interference or inhibitory interference. We therefore interpret Experiments 3 and 4 as support for the claim that reflexive-antecedent dependencies that are mediated by raising or control are processed more slowly and are more susceptible to interference than the co-argument dependencies when there is a distractor. Below, we outline a possible sequence of events that, while admittedly speculative, might explain how our raising and control sentences are affected by interference. For expository reasons, we focus on the *accessible-mismatch inaccessible-match* condition for Nominal Control in Experiments 3, as exemplified in (13): but analogous remarks also apply to Experiment 4.

13 **Nominal Control: Accessible mismatch, Inaccessible Match**
  Mary's agreement with Tom Ø to be kind to himself was surprising to everyone.

As discussed in the introduction of this paper, in (13), we assume that the control dependency is initially formed around the point where *to be kind* is read. Note that the retrieval is effectively triggered by a null element (i.e., Ø), so the retrieval cue cannot include gender information, so this retrieval is not expected to have been affected by gender-based interference. It is not possible to measure interference at this early point in the sentence with our design (and indeed, the experiment was not designed to detect this). In fact, our experiments investigated a second retrieval event, related to the processing of the reflexive, but so far, we have not discussed this second event in any detail. Accordingly, we now sketch a possible account, based on our experimental results.

In (13), we assume that, following the initial retrieval event at *to be kind*, the null element *Ø* is associated with information about its antecedent *Mary*, including the fact that the antecedent is female. At *himself*, the null element Ø is retrieved, and the gender incompatibility with the reflexive is registered, causing processing difficulty, and triggering a repair process. During the repair process, a new retrieval process is launched for Ø to find its antecedent. This now includes a male gender cue due to the fact that *himself* is male. It is at this point that *Tom* can be mis-retrieved as the antecedent of Ø, leading to processing facilitation. Note that, in order for this mis-retrieval to occur in the way that we have suggested, it would have to be possible for the reflexive to use gender as a retrieval cue (contra Dillon et al., 2013), as least during the repair process.

An alternative to the above account is that the interference that we observe in sentences like (13) is driven entirely by a repair process involving the reflexive-antecedent dependency in response to the gender mismatch, without a new control-related retrieval being launched. Thus, for example, the error at the reflexive might reduce confidence in the structural encoding, increasing sensitivity to other gender-matching items in the sentence. However, such an account would still need to explain the apparent lack of interference in the direct reflexive-antecedent dependencies examined in Experiment 1. In other words, if the reflexive triggered an error-based retrieval (without invoking control or raising dependencies) in Experiments 3 and 4, then why did it not also trigger an analogous error-based retrieval in Experiment 1? While this may potentially be due to other differences between the stimuli of Experiment 1 and the other experiments, we believe that the most likely reason is the fact that the relation between the reflexive and its antecedent is direct in Experiment 1, but mediated by control (or raising) in experiments 3 and 4, and that the control (or raising) dependency plays a role in the observed interference.

A question for future research is whether indirect dependencies (such as the ones that we examined in Experiments 3 and 4) are in general more prone to interference than direct dependencies (such as the one that we examined in Experiment 1).

## Author contributions

PS Supervised the running of the experiments, conducted the statistical analyses, and drafted the article. NK Supervised the creation of stimuli, and participated in writing the article. Both authors contributed equally to the planning of the research, and to the theoretical interpretation of the results.

### Conflict of interest statement

The authors declare that the research was conducted in the absence of any commercial or financial relationships that could be construed as a potential conflict of interest.

## Footnotes

^1^See Van Dyke (2007) and Gordon et al. (2006) for examples of inhibitory interference involving other types of dependencies. Gordon et al. (2006) argue that inhibitory similarity-based interference reflects feature-overlap or feature overwriting in the encoding stage, rather than multiple cue-overlap in retrieval.

^2^In addition to facilitatory interference and inhibitory interference, one other interference profile has been reported in the literature: in a study on the resolution of Spanish dependencies involving *otro* (similar to English “another”), Martin et al. (2012) reported processing disruption for grammatical dependencies where a distractor mismatched the retrieval cue.

^3^We used nominal control rather than verbal control because nominal control gives a wide range of control predicates that can be used in the *giver*-control condition (e.g., *vow oath*, *promise*). For verbal control, in contrast, there are very few control verbs that can be used in an analogous way.

^4^Technically, *John* is substituted into the subject position of the elementary tree headed by *kind*, and the elementary tree headed by *seems* is adjoined (i.e., inserted) into the elementary tree headed by *kind*.

^5^Co-argumenthood is an important notion in certain theoretical treatments of anaphoric binding (see Reinhart and Reuland, 1993, for a well-known example of such a theory).

^6^However, we acknowledge that the extra complexity of the control nominal dependency might not necessarily result in slower access. As pointed out by a reviewer, it is possible that the richer lexical information would in fact make access faster.

^7^The stimuli for all experiments are available in the Supplemental Material.

^8^The position of the control nominal in the sentence is different in Experiment 3 from Experiment 2. This is because Experiment 2 needed to use a sentence frame that allowed a comparison of nominal control with raising, while Experiment 3 only used nominal control, so could use a more naturally suited sentence frame.

^9^An elementary tree can be thought of as a lexically-stored extended projection of a head word.

^10^The XTAG grammar does not treat nominal control, but we assume that the analysis would be analogous to that of verbal control.
